# Advanced Nanocellulose-Based Materials: Production, Properties, and Applications

**DOI:** 10.3390/nano12030431

**Published:** 2022-01-27

**Authors:** Carmen S. R. Freire, Carla Vilela

**Affiliations:** CICECO—Aveiro Institute of Materials, Department of Chemistry, University of Aveiro, 3810-193 Aveiro, Portugal; cfreire@ua.pt

Natural polymers, such as polysaccharides and proteins, are being extensively utilized as substrates to create advanced materials [1,2,3,4]. Within the vast portfolio of natural polymers, bacterial nanocellulose (BNC), cellulose nanofibers (CNFs), and cellulose nanocrystals (CNCs), viz. the three nanometric forms of cellulose, are currently at the spotlight in numerous fields of modern science and technology [5,6,7]. The eco-friendly nature, peculiar features and multiple functionalities of these nanoscale cellulosic substrates are being investigated to engineer advanced nanocomposites and nanohybrid materials for application in manifold domains, such as mechanics, optics, electronics, energy, environment, biology, and medicine.

This Special Issue of *Nanomaterials* entitled Advanced Nanocellulose-Based Materials: Production, Properties, and Applications, brings together a compilation of original research and review contributions from world-leading scientists working with nanocellulose. Hence, this Special Issue contains a collection of one review paper about the characterization of cellulose nanomaterials [8] and eight research papers focused on the use of BNC [9,10,11], CNFs [12,13,14,15], and CNCs [16] as reinforcements in composites [13,14,15] and to produce ion-exchange membranes for fuel cells [9], patches for tissue engineering and wound healing [10,11], and nanosystems or nanocarriers for cancer treatment [15,16].

Under the title *Recent Progress on the Characterization of Cellulose Nanomaterials by Nanoscale Infrared Spectroscopy*, Zhu et al. [8] reviewed the latest advances in the applications of current state-of-the-art nanoscale infrared spectroscopy and imaging techniques, namely atomic force microscope-based infrared spectroscopy (AFM-IR) and infrared scattering scanning near-field optical microscopy (IR s-SNOM), to characterize cellulose nanomaterials. As stated by the authors, AFM-IR and IR s-SNOM are two techniques for compositional analysis and chemical mapping at the nanoscale spatial resolution that can also deliver insightful information on mechanical, thermal, and electrical properties for cellulose nanomaterials [8].

The study by Vilela et al. [9] demonstrated the feasibility of combining BNC (i.e., a microbial exopolysaccharide) with a water-soluble anionic sulfonated lignin derivative (i.e., lignosulfonates) and a natural crosslinker (i.e., tannic acid) to produce freestanding homogeneous membranes with good mechanical performance (maximum Young’s modulus of ca. 8.2 GPa) and moisture-uptake capacity (ca. 78% after 48 h) and a maximum ionic conductivity of 23 mS cm^−1^ (at 94 °C and 98% relative humidity). Even though the conductivity values achieved are comparable or higher than other fully biobased ion-exchange membranes reported in the literature, they are still two orders of magnitude lower than the standard commercial Nafion^TM^ ionomer currently in use in fuel cells. Still, and according to the authors, this study might contribute to the long and laborious path towards the development of eco-friendly conducting separators, especially through the exploitation of surplus raw materials from agricultural and industrial by-products [9].

Similarly interesting is the investigation of Kutová et al. [10] that examined the effect of the drying method (air-drying or freeze-drying) and subsequent argon plasma modification of BNC on the adhesion and growth of human keratinocyte cells (HaCaT cell line). The air-dried method followed by argon plasma modification yielded a BNC membrane with lower porosity (total pore volume (V_p_) = 0.142 ± 0.008 cm^3^ g^–1^) and specific surface area (S_BET_ = 140.5 ± 4.8 m^2^·g^−1^) when compared with the freeze-dried and argon plasma-modified BNC (V_p_ = 0.308 ± 0.015 cm^3^ g^–1^ and S_BET_ = 156.3 ± 1.5 m^2^·g^−1^). The growth and adhesion of HaCaT cells, when BNC was air-dried or freeze-dried, was markedly improved by the argon plasma modification. As stated by the authors, the surface modification rendered BNC a good support for the adhesion, growth, and viability of human HaCaT keratinocytes and thus can be considered highly promising materials for skin tissue engineering and wound healing [10].

In a different study, Fonseca et al. [11] reported the preparation and characterization of multilayered patches composed of oxidized BNC membranes (prepared via TEMPO (2,2,6,6-tetramethylpiperidin-1-yl)oxyl)-mediated oxidation of BNC) loaded with dexpanthenol (DEX), an active ingredient used in topical products for the treatment of dermatological conditions (e.g., wound healing). Spin-assisted layer-by-layer (LbL) assembly was employed to coat the DEX-loaded BNC membranes with alternate layers of chitosan and alginate polyelectrolytes, yielding multilayered patches with up to a total of 21 layers. The patches retarded the release of the DEX from the three-dimensional nanostructure of BNC, depending on the number of layers (ca. 95% after 16 h for the DEX-loaded BNC membrane versus ca. 65% after 90 h for the DEX-loaded BNC membrane with 21 layers). Furthermore, the multilayered patches inhibited the growth of *Staphylococcus aureus* (owing to the antimicrobial action of chitosan), were non-cytotoxic to human keratinocyte cells (HaCaT cell line) and showed good migration results in the wound healing scratch assay, suggesting the pertinence of these systems in the treatment of skin wounds [11].

In the domain of CNFs, and as highlighted in the research by Jonasson et al. [12], the lignin content influenced the one-pot direct TEMPO-oxidative ) nanofibrillation of wood cell wall. In fact, wood from poplar trees with high lignin content (30.0 wt.%) was more easily fibrillated as revealed by the higher nanofibril yield (68% and 45%) and suspension viscosity (27 and 15 mPa·s) than low lignin wood (17.4 and 19.7 wt.%). Moreover, the surface area (114 m^2^·g^−1^) and pore size (5.0 nm) of the oxidized high lignin wood were higher than those of low lignin wood (76 m^2^·g^−1^ and 4.4 nm, respectively), which means that porosity is a factor that can also beneficially impact the isolation of CNFs from wood [12].

Another original work was reported by Nissilä et al. [13], who investigated the use of ice-templated CNF filaments as a reinforcement material in epoxy resin-based composites. The authors successfully manufactured cellulose nanocomposites with an oriented structure and a strong fiber–matrix interface by preparing unidirectionally aligned CNF-filament mats via ice-templating followed by chemical vapor deposition to obtain silane-treated CNF filaments. The impregnation of these mats with a bio-epoxy resin through vacuum infusion originated composites with 18 wt.% fiber content oriented along the freezing direction of the ice crystals, which translated into composite materials with improved mechanical performance (storage modulus) up to 2.5-fold [13].

Xue et al. [14] prepared high-strength regenerated cellulose composite fibers reinforced with CNFs and nanosilica (nano-SiO_2_). The incorporation of 1% of CNFs and 1% of nano-SiO_2_ into a cellulose solution of an ionic liquid, 1-allyl-3-methylimidazolium chloride (AMIMCl), improved the mechanical properties of the regenerated composite cellulose fibers by 47.46%. In addition, the viscosity of the cellulose/AMIMCl mixtures (with or without CNFs and nano-SiO_2_) was characteristic of pseudoplastic fluids, and the storage and loss moduli (elasticity and viscous moduli) decreased with the temperature, representing a reduction in viscoelasticity [14].

Equally interesting is the study of Yusefi et al. [15], which investigated the use of the LbL methodology to assemble nanocomposites (diameter below 50 nm) of chitosan reinforced with CNFs for the encapsulation of an anticancer drug, i.e., 5-fluorouracil. The nanocomposites, with a pH-responsive behavior and non-cytotoxicity towards the human colonic CCD112 cell line, exhibited a drug encapsulation efficiency of ca. 86% and were able to eliminate (as intended) 56.42 ± 0.41% of the human colorectal cancer HCT116 cells at a concentration of 250 µg mL^–1^, showing their potential as nanocarriers of anticancer drugs [15].

Lastly, Pinto et al. [16] developed CNC nanosystems functionalized with a chitosan derivative holding both targeting (folic acid) and imaging (fluorescein isothiocyanate) functions. The simple and environmentally friendly method of physical adsorption was adopted, considering the opposite charges of CNCs with a zeta (ζ)-potential of −11.5 ± 0.7 mV at pH 7 and the chitosan derivative with a ζ-potential value of +51.9 ± 3.6 mV at pH 3. The CNCs nanosystems demonstrated improved internalization (up to five-fold) in human breast adenocarcinoma cells (MDA-MB-231) and a potential anti-proliferative effect, hinted by exometabolomic analysis, suggesting the applicability of these nanosystems in active targeted cancer therapy [16].

Overall, the nine papers in this Special Issue of *Nanomaterials*—belonging to the section Nanocomposite Materials—addressed the exploitation of nanocellulosic substrates to manufacture advanced nanocomposites and nanohybrid materials for both biomedical (e.g., wound healing [11] and anticancer treatment [15,16]) and technological (e.g., fuel cells [9]) applications.
